# Relationship between Spatial Working Memory Performance and Diet Specialization in Two Sympatric Nectar Bats

**DOI:** 10.1371/journal.pone.0023773

**Published:** 2011-09-09

**Authors:** Mickaël Henry, Kathryn E. Stoner

**Affiliations:** 1 Institut National de la Recherche Agronomique, UMR 406 Abeilles & Environnement, Site Agroparc, Avignon, France; 2 Centro de Investigaciones en Ecosistemas, Universidad Nacional Autónoma de México, Morelia, México; 3 Department of Biological and Health Sciences, Texas A & M University-Kingsville, Kingsville, Texas, United States of America; University of Western Ontario, Canada

## Abstract

Behavioural ecologists increasingly recognise spatial memory as one the most influential cognitive traits involved in evolutionary processes. In particular, spatial working memory (SWM), i.e. the ability of animals to store temporarily useful information for current foraging tasks, determines the foraging efficiency of individuals. As a consequence, SWM also has the potential to influence competitive abilities and to affect patterns of sympatric occurrence among closely related species. The present study aims at comparing the efficiency of SWM between generalist (*Glossophaga soricina*) and specialist (*Leptonycteris yerbabuenae*) nectarivorous bats at flowering patches. The two species differ in diet – the generalist diet including seasonally fruits and insects with nectar and pollen while the specialist diet is dominated by nectar and pollen yearlong – and in some morphological traits – the specialist being heavier and with proportionally longer rostrum than the generalist. These bats are found sympatrically within part of their range in the Neotropics. We habituated captive individuals to feed on artificial flower patches and we used infrared video recordings to monitor their ability to remember and avoid the spatial location of flowers they emptied in previous visits in the course of 15-min foraging sequences. Experiments revealed that both species rely on SWM as their foraging success attained significantly greater values than random expectations. However, the nectar specialist *L. yerbabuenae* was significantly more efficient at extracting nectar (+28% in foraging success), and sustained longer foraging bouts (+27% in length of efficient foraging sequences) than the generalist *G. soricina*. These contrasting SWM performances are discussed in relation to diet specialization and other life history traits.

## Introduction

The past decade has witnessed the rise of a new approach to animal behaviour, called cognitive ecology [1]. Cognitive ecology is concerned with the process of decision-making in animals in their environment, and its consequences for reproductive success. Cognitive ecologists interpret animals' cognitive ability as an adaptive response to the natural selection pressure exerted by their environment. The logical reasoning behind this is that better cognitive abilities enhance animals' fitness by improving their ability to acquire food, escape predators or choose mates ([2] and other references therein).

Memory is recognised as one the most influential cognitive traits in evolutionary processes [2]. In particular, animals relying on spatially scattered food resources, such as seed-caching birds, may develop better spatial memory skills than conspecific individuals with different foraging habits [3]. Spatial memory consists of the mental storage of spatial coordinates of past visited locations. This information may be used for subsequent relocation of roosts and high-quality food sources [4], or avoidance of areas with high predation risks [5].

Two types of spatial memory can be distinguished: spatial reference memory and spatial working memory. Also termed long-term and short-term memory, respectively [6], reference and working memories differ by the persistence time of the information they process. Spatial reference memory stores spatial information on a virtually permanent basis, while spatial working memory only stores temporarily useful information [6]. Animals that forage on static food sources of variable quality, like temporarily available fruit crops or nectar sources depleted by competitors, rely heavily on spatial working memory. Beyond the location of a given food source, they must retain the information of its state (e.g. depleted or not) at the time of the last visit. This information is unstable and updated at each subsequent visit.

The use of spatial working memory (SWM) has been investigated in a wide range of frugivorous, granivorous and nectarivorous animals, including honeybees [7], birds [8], rats [9] and bats [10]. SWM can be evidenced using radial-arm maze experiments whereby animals have to remember a pathway through a suite of corridor bifurcations to eventually reach food rewards. In a more naturalistic approach, other studies have used open-field maze experiments [8] where animals are free to navigate among scattered rewards. In the later approach, SWM performance is indicated by the animals' ability to avoid past-visited places devoid of rewards, as compared with random walk simulations.

Although SWM has been evidenced in various species taken individually, few studies have attempted to document its variations across environments or closely related species [5], [11], [12]. Yet, thorough comparisons among populations in different environments or closely related species is a crucial step for a better understanding of which cognitive traits are selected by evolutionary processes and how cognitive plasticity relates to fitness [2]. Individuals from the same species but exposed to environments with different selection pressure may display different memory performance [5]. Likewise, unequal memory performance among sympatric species may help explain contrasting competitive abilities or spatial segregations among habitats [11].

The present study aims at comparing the efficiency of spatial working memory in two sympatric nectar bats (Phyllostomidae: Glossophaginae). Bats in general [13], [14], and phyllostomid nectar bats in particular [10], [12], [15] display excellent spatial memory. Nectar bats have probably evolved such cognitive abilities in response to the need to repeatedly visit and handle huge quantities of flowers (up to 1000 [16]) to fulfil their daily energy requirement from small nectar rewards. One of the most striking pieces of evidence of their high-performance SWM is exemplified by their capacity to remember the location of individual flowers they have visited and depleted in recent foraging sequences, in order to avoid subsequent non-rewarding revisits [10]. Captive individuals of Pallas's Long-tongued bat *Glossophaga soricina* exposed to an array of 64 artificial flowers succeeded in exploiting in a single foraging sequence up to 40 flowers without a substantial decrease in foraging success due to revisiting previously depleted flowers.

Unequal SWM among nectar bat species may result in different nectar intake rates at flower patches (e.g. flowering trees), and may in turn lead to asymmetric competitive abilities. This has the potential to determine, to a large extent, the spatiotemporal pattern of foraging activity in sympatric species through mechanisms of optimal foraging. For instance, inferior competitors may prefer foraging in areas where superior competitors are fewer, in order to reduce competition pressure for nectar exploitation. The objective of the current study is to compare SWM performance at artificial flower patches between two phyllostomid nectar bats found sympatrically within part of their range, namely *Glossophaga soricina* and *Leptonycteris yerbabuenae*. Using an experimental setup coupling artificial flowers and infrared video recordings, we tested whether (i) both species effectively rely on SWM during short foraging sequences and (ii) the SWM performance differs between the two species. As observed in previous studies [10], we expected *G. soricina* to rely on a SWM system to improve foraging efficiency at the flower patch level. However, we further expected *L. yerbabuenae* to display even greater SWM performance given its specialised nectarivore habits, as opposed to the generalist habits of *G. soricina* that may complement its diet with fruits or insects during food shortage periods [17]–[20]. *L. yerbabuenae* also exhibits a proportionally more elongated rostrum than *G. soricina*, a common adaptation of flower-visiting bats.

## Materials and Methods

### Ethics statement

All experimental procedures in this study adhered to the laws of the Mexican Government (SEMARNAT, Secretaría de Medio Ambiente y Recursos Naturales) and follow the guidelines outlined by the Oficina de Fauna Silvestre, Mexico (SGPA/DGVS Permit 3644 to KES). Although our institution, Universidad Nacional Autónoma de Mexico (UNAM), does not yet have an Institutional Review Board or a similar governing body of ethics, this project and experimental protocol was approved by the institutional authorities from UNAM (Project PAPITT IN226007).

### Study species

Bats were collected in the region of the Chamela-Cuixmala Biosphere Reserve in the central Pacific coast of Jalisco, Mexico (ca. 19°22′–19°35′N, 104°56′–05°03′W). The principal vegetation in this area is tropical dry forest [21]. Five nectarivorous bats are known to occur in this region: *Choeroniscus godmani*, *Glossophaga commissarisi*, *G. soricina*, *Leptonycteris yerbabuenae*, and *Musonycteris harrisoni* [22]. We performed experiments on the two most common species of the region: Saussure's long-nosed bat *Leptonycteris yerbabuenae*, a nectar specialist, and the Long-tongued bat *Glossophaga soricina*, a nectar generalist (both species Phyllostomidae: Glossophaginae).

### Bat capture and housing

Experiments were carried out on 8 individuals of each species, using only non-reproductive adult males to avoid any confounding effect of gender or undetected pregnancy. All individuals were collected within 3 days in March 2007. We used mist nets to capture bats close to the entrance of known roosts. After captures, bats were transferred to the laboratory at the Centro de Investigaciones en Ecosistemas, Universidad Nacional Autonoma de Mexico. Bats were housed in a dark room within species specific colonies, with temperature (26°c) and humidity (70–75%) set close to field conditions. Bats were fed a maintenance diet based on soy milk, cow's milk, and fruit, and supplemented with a vitamin and mineral mix (NEKTON-Plus; Pforzheim, Germany) [23]. Body mass, wing membrane elasticity and hair condition of all bats was monitored daily. All bats maintained constant body mass and appeared healthy while in captivity. All experiments were conducted during the 3 months following capture and bats were thereafter released at the capture site.

### Experimental setup

**Figure 1:**
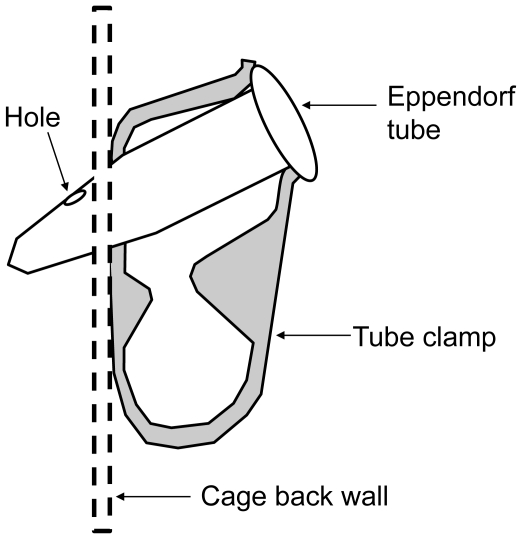
Profile view of nectar feeder composed of a 1.5-ml Eppendorf tube attached to the back of the cage by a white plastic tube clamp. The bottom hole permits the replenishment of the feeder from outside the cage with a micropipette.

We simulated a flower patch using 25 artificial flowers placed in a 5 by 5 square grid with 20 cm between flowers. The flower patch was centred on the back wall of a 1.5-m cubic flight cage. Artificial flowers (Figure 1) were feeders composed of a 1.5-ml Eppendorf tube maintained against the cage wall by a white plastic ratcheting tube clamp (Small Parts, Inc, Miramar, FL). The tubular shape of feeders offered bats valuable echo-acoustic cues to locate the tube opening. The feeder tube was oriented opening upward, with an angle of 45° and could be replenished from outside the cage through a small hole at 1 cm from the bottom (Figure 1). Bat visits to feeders were monitored using an infrared digital video camera (Digital CCD Camera, Innovative technology, Model H.R. IR camera) recording on a continuous basis at a rate of 30 images per second.

### Habituation

Bats were habituated to foraging in the experimental environment during two consecutive nights in conspecific groups of 15 individuals. All 25 feeders were filled up with maintenance diet during the first night and the experimental sucrose solution (see below) was used during the second night. Additional maintenance diet was provided *ad libitum* in buckets both at the beginning and at the end of the night to ensure individuals could meet their daily food requirements.

### Experimental procedure

Experiments started after the two nights of habituation. Bats were processed individually to measure their performance at foraging in the feeder patch. Feeders received small volumes of 20% wgt sucrose solution to mimic natural nectar rewards [20]. We used different solution volumes for the two species to account for their different food intake capacities and therefore possibly different satiation thresholds. *Leptonycteris yerbabuenae* is, on average, twice as heavy as *G. soricina* (in our study: 20.5–25.0 g vs. 8.6–11.5 g, respectively). Accordingly, in the course of preliminary experiments conducted with 15 individuals of each species, *L. yerbabuenae* consumed approximately twice as much 20% sucrose solution as *G. soricina* each night (35.5±7.1 g and 17.6±2.9 g, respectively). Therefore, we provided *L. yerbabuenae* with nectar rewards twice those of *G. soricina*, namely 40 µl and 20 µl per feeder, respectively. These volumes fit natural rewards of flowers found in the field [20] and appear as a good way to meet the different species' requirements. The nectar reward had to be small enough to be consumed by bats in a single visit, yet large enough so that we could visually determine whether they were consumed or not. The presence or absence of these quantities in the Eppendorf feeders could be visually evaluated.

Bats were tested individually in the artificial flower patch during trials of 15 min. This time lapse encompassed twice the average time necessary for an individual to consume most of the available nectar in the patch, based on habituation trial observations (median time for 50% nectar removal = 3.3 min., mean = 5.7±2.9 min., n = 16 trials). It also approximates the smallest refill intervals reported for natural flowers [10]. At the end of each 15-min trial, a weak light was turned on in the room during 5 minutes to give bats a signal that the current trial was over. All depleted feeders were then visually identified and replenished from outside the cage using a micropipette before starting over the next trial; bats did not forage during this replenishing period. Experiments continued until six successful trials were achieved per individual. We considered a 15-min trial successful when bats would make at least 25 feeder visits (i.e. an average of one visit per feeder in the patch). We estimated from habituation trials that when 17 feeders were depleted, bats had completed foraging sequences of at least 30 visits in nearly all cases. In other words, it took bats' approximately 30 total visits (rewarding visits and non-rewarding revisits) to achieve 17 rewarding visits. Therefore, we used the average of 17 emptied flowers as an indication that the minimum objective of 25-visit sequences was fulfilled and the trial was successful. Once bats completed six successful trials, typically within 2–3 hours, they were released in their respective conspecific colony where maintenance diet was provided *ad libitum*.

### Data collection

Video recordings were inspected at reduced speed to search for all bat visits to feeders. Visits were typically a 0.2-s to 1-s hovering flight in front of feeders, or sometimes a short landing on the feeder structure. Analyses were restricted to the first 25-visit sequence of each successful trial, therefore totalling 2400 visit data points (25 visits × 6 trials × 8 individuals × 2 species). Each visit was characterised by its rank in the visit sequence (from 1 to 25), its position in the patch (row and column), its success (1 for rewarding first visit, 0 for non-rewarding revisit), and time interval since last visit to this same flower (±1 s). Two visits performed at the same feeder in less than 1 s were considered as a single visit.

We assessed observational errors – recording a visit when the individual did *not* drink the nectar – by comparing the list of feeders scored as visited during the video footage, against those that were not emptied at the end of trials. On average, only 3.5% of the visit records scored from videos was incorrectly identified (i.e. false visits with *no* nectar consumption). This bias was similar between species and was unlikely to affect our ability to detect interspecific variations in foraging success. Furthermore, by referring to non-emptied feeders as emptied, false visit records actually lead to a slight underestimation of foraging success. As such, recording false visits is a conservative bias regarding our hypothesis of SWM, which is evidenced by a greater foraging success than random expectations.

### Evidence of spatial working memory

Evidence of bats using SWM is found if foraging success is significantly greater than expected by random visit orders. Random expectations were simulated by computing 1000 sequences of 25 visits randomly sampled with replacement from the 2400 observed visit dataset. Observed foraging success greater than the simulated 95% confidence interval of random expectations would support the hypothesis of an effective SWM.

### Interspecific variations in spatial working memory

We used Generalized Linear Mixed Effect models (GLMMs) to test the hypothesis that SWM, and hence the foraging success, varies between species. Foraging success was defined as the probability of obtaining a nectar reward at a given flower visit. GLMMs allowed us to handle the binary (0 vs. 1) visit success data using a binary error distribution, and to further account for the autoregressive nature of the repeated measures on the same individuals and within the same trials. In particular, the probability of visit success decreases as visit rank increases during a trial. The visit rank effect was controlled for by introducing into models the visit rank as a random continuous variable. Likewise, individuals were introduced as a random grouping variable. The first visit of each trial was excluded from analyses as it is invariably a rewarding visit. Models were fitted using the penalized quasi-likelihood approximation for further assessing the significance of species effect by means of a likelihood ratio test using the lme4 library [24] in R 2.8.1 [25].

### Interspecific variations in foraging pattern

**Figure 2:**
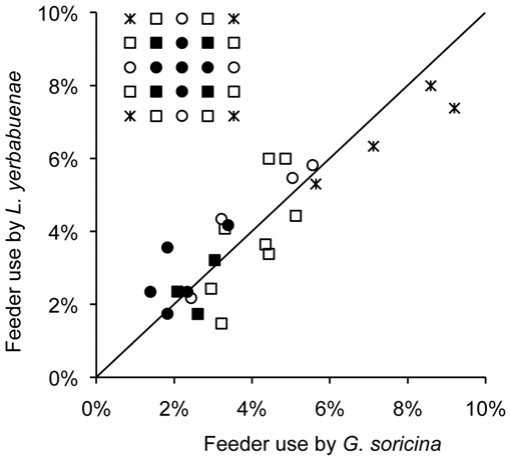
Relative use of flowers by the two bat species. The upper left diagram shows the relative position of the 25 different feeders. The line of slope one indicates expected values for equal use of feeders by the two species. Feeders' use by *G. soricina* and *L. yerbabuenae* are tightly correlated (Pearson = 0.89, P<0.001).

Possible differences in mean foraging success between species may be a consequence of unequal SWM per se, or of distinct foraging patterns within the feeder patch. Foraging pattern refers to the distribution of foraging activity in space and time. In particular, shorter time intervals between visits may improve foraging success owing to the recency of the information stored in memory. Likewise, visiting nearby feeders in a systematic manner may be a more efficient foraging pattern than choosing more distant feeders every next visit. We investigated this issue by assessing interspecific differences in distance (cm) and time (s) intervals between successive feeder visits, using models analogous to those described above. As distance and time intervals (log-transformed) were normally distributed, we favoured Linear Mixed Effect models (LME), using the maximum likelihood method and specifying a Gaussian error distribution. Visit rank and individuals were kept as random effects. However, in the case of inter-feeder distance, all feeders do not share the same range of distance possibilities. Feeders in peripheral positions have larger distance values than feeders closer to the centre of the patch. To account for this positional effect, we classified feeders into five symmetrical position groups that were afterward introduced as an additional grouping level in the model (see diagram in Figure 2 for an illustration). Among the 16 peripheral feeders, we defined three positional groups: the four corner feeders, the four median feeders, and the eight intermediate feeders. Among the nine inner feeders, we identified two positional groups: the four corner feeders and the five median feeders (including the central one).

## Results

Bats did not use the 25 feeders in equal proportions, but the two species showed similar preferences, judging from the strong correlation between the relative use of feeders by the two species (Figure 2). Under the hypothesis of a uniform use, each feeder should account for 1/25 = 4% of the visits. However, feeders in peripheral positions were actually used more often (relative use >4%, and up to 6–8% for corner feeders, Figure 2), while feeders in inner positions were used less often (2–4%). Furthermore, individuals rarely chose adjacent feeders in successive visits, usually shifting to feeders located two to three positions away (average shift = 1.6±1.2 lines and 2.1±1.4 columns).

### Evidence of spatial working memory

**Figure 3:**
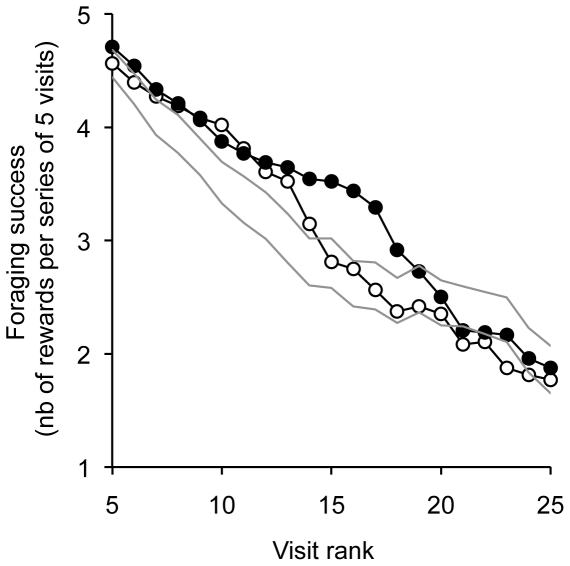
Foraging success of bats in an array of 5×5 feeders. Closed circles: *L. yerbabuenae*; open circles: *G. soricina*; grey lines: 95% confidence interval for the null model. *Leptonycteris yerbabuenae* is up to 25–28% more efficient than *G. soricina* (visits #15 to #19).

Both species showed evidence of SWM. Their mean foraging success lies above the 95% confidence interval of random expectations until they made >14 visits out of the 25 feeders (Figure 3). In other words, foraging sequences could contain >14 decisions with an overall significant use of spatial memory.

### Interspecific variations in spatial working memory

*Leptonycteris yerbabuenae* was able to sustain longer foraging sequences (up to 19 feeder visits) with greater foraging success than random expectations (Figure 3). This is about 27% longer than *G. soricina* whose foraging success drops into the random range at the 15^th^ feeder visit. Between visits #13 and #17, the nectar intake rate of *L. yerbabuenae* was up to 23–28% greater than that of *G. soricina*.

Yet, GLMMs indicate that foraging success did not vary significantly among species over the first 25 visits of trials (Table 1). However, given the rather irregular decrease of mean foraging success during trials (Figure 3), we suspected different mechanisms would act upon foraging success on different sections of the 25-visit trials. We therefore repeated the analyses separately for each sequence of five consecutive visits. Five-visit sequences were the finest partitioning option the dataset could reasonably afford considering statistical power. It allowed reaching the sample size threshold of 30 data points per individual (5 visits×6 repetitions), which is usually recommended for parametric statistics. After splitting up the dataset, we found *L. yerbabuenae* had a greater foraging success than *G. soricina* over most of the 25 visits – i.e. a greater probability of being rewarded at a given feeder visit (see positive estimates in Table 1), but this difference was effectively significant for a single sequence, namely visits #12 to #16, conforming to the trends depicted by curves in Figure 3.

**Table 1:** Results of GLMM analyses testing for differences in foraging success between bat species.

Visit sequences	*N*	Intercept	Estimate	L-ratio Chi-square on 1 *df*	*P*
All visits (2 to 25)	2304	0.532±0.038	0.211±0.054	2.36	0.124
Visits 2 to 6	480	2.18±0.062	0.338±0.094	0.939	0.332
Visits 7 to 11	480	1.27±0.065	−0.099±0.091	0.195	0.659
**Visits 12 to 16**	**480**	**0.172±0.064**	**0.617±0.094**	**10.02**	**0.001**
Visits 17 to 21	480	−0.441±0.078	0.142±0.111	0.380	0.537
Visits 22 to 25	384	−0.533±0.072	0.044±0.102	0.044	0.833

Positive estimates indicate average foraging success is greater in *L. yerbabuenae* than in *G. soricina*. *N*: sample size; *P*: Chi-square probability.

### Interspecific variations in foraging pattern

The greater foraging success of *L. yerbabuenae* in visits #12 to #16 could not be explained by significantly shorter distances, nor by significantly shorter time lapses between successive feeder visits (Table 2). The negative estimate in Table 2 indicates that *L. yerbabuenae* chose feeders closer to each other for successive visits, compared to *G. soricina*. The mean difference in distance lag was however small and remained non-significant (1.91-cm distance difference, representing only 3.1% of the mean, *p* = 0.452). Likewise, *L. yerbabuenae* visited feeders at a slightly faster rate compared to *G. soricina* (mean rate = one visit every 7.6 s and 6.6 s, respectively), but this difference was statistically non-significant (*p* = 0.387). Similarly, distance and time lapses remained statistically equivalent (or nearly so) between the two species during all other visit sequences.

**Table 2:** Results of LME analyses testing for differences in inter-feeder distance and time lapse between successive visits for the two bat species.

	*N*	Intercept	Estimate^a^	L-ratio Chi-square on 1 *df*	*P*
Distance (cm)					
All visits (2 to 25)	2304	58.13±1.53	−3.27±2.16	2.25	0.133
Visits 2 to 6	480	55.62±2.04	−2.26±2.88	0.592	0.442
Visits 7 to 11	480	60.41±2.02	−3.83±2.83	1.73	0.189
Visits 12 to 16	480	61.27±1.79	−1.91±2.53	0.566	0.452
Visits 17 to 21	480	61.05±2.27	−4.14±3.18	1.67	0.196
Visits 22 to 25	384	60.01±2.32	−5.86±3.29	3.09	0.079
Time lapse (log_10_[s])					
All visits (2 to 25)	2304	0.911±0.031	−0.080±0.043	3.02	0.082
Visits 2 to 6	480	0.869±0.041	−0.070±0.057	1.30	0.254
Visits 7 to 11	480	0.833±0.033	−0.008±0.047	0.027	0.870
Visits 12 to 16	480	0.882±0.051	−0.063±0.072	0.748	0.387
**Visits 17 to 21**	**480**	**0.934±0.038**	**−0.112±0.054**	**3.87**	**0.049**
Visits 22 to 25	384	0.943±0.037	−0.083±0.052	2.36	0.125

a Negative estimates indicate average distance and time lapse are shorter in *L. yerbabuenae* than in *G. soricina*. Note the intercept and estimates are in *cm* and *log_10_-s* for distances and time lapses, respectively. *N*: sample size; *P*: Chi-square probability.

## Discussion

We have shown in this study that both species use SWM to improve their foraging success in flower patches, i.e. they have developed abilities to remember the location of visited flowers during past visit sequences. However, the nectar specialist *L. yerbabuenae* is more efficient at this task, showing both greater foraging efficiency and longer efficient foraging sequences. We cannot attribute the observed greater foraging success of *L. yerbabuenae* to either spatial or temporal allocation of foraging activity, as we found no significant differences between these attributes in the two species. Therefore, a more efficient SWM in *L. yerbabuenae* is the most parsimonious explanation to account for its greater foraging success within the artificial flower patch.

### Comparison with previous studies

Our observations on *G. soricina* mostly conform to those reported on the same species in earlier studies [10]. First, individuals displayed similar behavioural patterns when visiting feeders, with short hovering flights generally <1 s in duration, and they successively visited feeders in non-adjacent positions. Second, individuals had disproportionate preferences for the feeders located in peripheral positions, especially corners, while they visited much less often feeders in central positions (Figure 2). The exact same behavioural bias was reported by Winter and Stich (Figure 5b in [10]). We think relocating flowers in peripheral positions may be facilitated by the possibility for bats to rely on visual landmarks. Previous work has shown *G. soricina* to use the relative configuration of feeders as a cue for orientation [12], [26]. In our experimental setup, the nine central feeders all displayed identical visual configuration with regards to the eight direct neighbours, making it more difficult for bats to visually discriminate among individual feeders. On the contrary, peripheral feeders differed from each other with only 3 or 5 neighbours located above, below, on the left or on the right. From a visual perspective, this variable-configuration possibly facilitates orientation by bats, and therefore may be preferred over the central constant-configuration. The third and most important similarity we found with Winter and Stich [10] is the use of a SWM process by *G. soricina* to improve foraging success.

### Evidence of spatial working memory

*Glossophaga soricina* individuals had a foraging success significantly greater than expected from random feeder visits. Individuals could perform up to 15 visits from 25 feeders until their average foraging success fell within the range of random expectations. Interestingly, this leads to a ratio of patch exploitation efficiency (15/25 = 60.0%) that closely matches the one reported by Winter and Stich [10] (40/64 = 62.5%). The later authors concluded the SWM performance of *G. soricina* appeared to surpass previous findings on other taxa. In our study we found that the more specialised nectar bat *L. yerbabuenae* showed an even greater SWM than its closely related counterpart *G. soricina*, with a corresponding ratio reaching 19/25 = 76%. Beyond the greater storage capacity of its SWM, *L. yerbabuenae* also displays better storage reliability. Its average foraging success surpassed that of *G. soricina* along nearly the entire 25-visit sequence (Figure 3). The relative difference in foraging success between species peaked at 25–28% in visits #15–19. At this point, the foraging success of *L. yerbabuenae* was 18–22% greater than the upper 95% confidence limit for random expectations.

We could not find any alternative explanation for the difference between *G. soricina* and *L. yerbabuenae*, other than unequal SWM per se. In particular, no significant difference was found between species in terms of spatial and temporal allocation of foraging activity within the artificial flower patch (Table 2). Yet, a consistent trend emerged that *L. yerbabuenae* visited feeders located on average closer to each other and with shorter time lapses, whatever the visit sequence we consider (see negative estimates in Table 2). However, average distance and time differences remained trivial compared to the 27% difference in length of efficient visit sequences and the 28% difference in average foraging success. Therefore, the foraging patterns of the two species do not appear sufficiently contrasting to alone account for the differences in foraging success without marked interspecific variation in SWM performance.

### Causes of interspecific variations in spatial working memory

One possible explanation for the better performance observed in *L. yerbabuenae* is that this species has evolved better SWM as an adaptation to its greater specialization level for floral resources. While *G. soricina* is a generalist nectarivore, i.e. with omnivorous habits depending on food availability [17], *L. yerbabuenae* heavily feeds on floral resources all year round [19]. Such a dietary specialisation likely could result in selection for greater SWM to assure individuals meet their dietary requirements. Since daily nectar production is variable and nectar is dispensed in extremely small rewards of only several µl to tens of µl at a time [27], this forces individuals to visit many flowers to meet their daily energy requirements, and to do so under a tight time schedule. High-performance SWM may be viewed as a means of counterbalancing this temporal constraint by fostering the rate of reward acquisition per time unit at flower patches. Following this scenario, it may be hypothesized that the onset of nectarivory in bats has resulted in the selection of individuals with greater SWM performance. The most recent molecular advances show that phyllostomid bats actually first evolved omnivory from insectivorous ancestors, following metabolic pre-adaptations [28]. After these pre-adaptations were achieved, further adaptations lead to nectarivory, such as rostrum and tongue elongation, hairlike papillae, teeth reduction and the ability to use hovering flight while ingestion. The latter adaptations may include better cognitive abilities compared with omnivorous species [14].

There are, however, alternative explanations for the greater SWM performance in *L. yerbabuenae*; beyond the nectarivore specialization hypothesis, SWM can be seen as an adaption to its greater body mass. *Leptonycteris yerbabuenae* is roughly twice as heavy as than *G. soricina* and consumes twice more nectar to meet daily energy requirements. It may have developed more acute cognitive abilities in response to the need for greater food intake. Another explanation includes the potentially high competition pressure for nectar exploitation around colonies. This highly gregarious species usually forms large colonies of thousands to tens of thousands of individuals in caves [19], [29], placing individuals under potentially intense competition in the vicinity. Competition may at least be most probable during seasonal food shortage periods, when the bulk of the population is forced to migrate several hundreds of kilometers northward [19]. *Glossophaga soricina*, on the contrary, roosts in small groups of several tens to hundreds of individuals only, and therefore may be less constrained by intraspecific competition at local scale.

All these tentative explanations for the selection of greater SWM by evolution are not mutually exclusive. They may be further explored by repeating SWM experiments with individuals from different geographical areas that have evolved under different conditions of competition pressure and food resource availability, or individuals from other nectarivorous species with different body size and/or dietary habits. In particular, other nectar specialists should be challenged, such as the Colima long-nosed bat *Musonycteris harrisoni* that has a remarkably elongated snout as a striking signature of its super-specialization on flowers [30], [31]. Likewise, SWM tests on the Lonchophylinae species should give interesting insights as this subfamily evolved nectarivory independently from Glossophaginae species within the phyllostomid bat family [28].

### Consequences of interspecific variations in spatial working memory

One potential outcome of unequal SWM is that species are unequal competitors and may display different spatial patterns of foraging activity. This statement is supported by some field data from our study area, where both species share the same keystone floral resources, mainly Bombacaceae trees of the genus *Ceiba*, and Agavacae and Cactacae species [19], [32], [33]. Behavioural observations at flowering *Ceiba grandiflora* have shown *L. yerbabuenae* and *G. soricina* are obviously engaged in a competitive process. Their respective visit frequencies at flowers are negatively correlated with each other [32]. But most interestingly, more detailed analyses revealed that visit frequencies of *G. soricina* decrease significantly as patch size increases (i.e. the number of open flowers in the tree, mean = 8.9±11.8, range = 1 to 52), while *L. yerbabuenae* visits followed an opposite trend (Figure S1). This statistical interaction between species and patch size suggests that (i) flower patches are more attractive to *L. yerbabuenae* when they offer more flowers, and (ii) *G. soricina* is more often observed at smaller patches where competition is potentially more relaxed. This can be further interpreted as a form of competitive exclusion of *G. soricina* by *L. yerbabuenae* that is more efficient at exploiting larger patches because of its larger size and greater performing SWM.

Many other behavioral aspects need to be considered in this perspective to bridge theoretical predictions from SWM experiments to observed spatial foraging patterns. These include, among others, trap-lining behavior at the inter-patch level, or territoriality in *G. soricina* [17] vs. group foraging in *L. yerbabuanae* [34]. In spite of the fact that our study cannot control for all of these parameters, our data clearly show that a cognitive approach to ecology of species interactions may offer new insights about the understanding of spatial foraging patterns.

## Supporting Information

Figure S1 **Visit frequencies of nectarivorous bats at individual** ***Ceiba grandiflora*** **flowers, as a function of total flower numbers in the trees.**
(PDF)
