# Supplementary material for: Relationship between Spatial Working Memory Performance and Diet Specialization in Two Sympatric Nectar Bats
Source: PLoS One. 2011 Sep 9;6(9):e23773. doi: 10.1371/journal.pone.0023773 (PMC3170290; doi:10.1371/journal.pone.0023773)
Supplement: Figure S1 — Visit frequencies of nectarivorous bats at individual Ceiba grandiflora flowers, as a function of total flower numbers in the trees. (PDF) [file pone.0023773.s001.pdf]

Supporting Information

Figure S1. Visit frequencies of nectarivorous bats at individual *Ceiba grandiflora* flowers, as a function of total flower numbers in the trees.

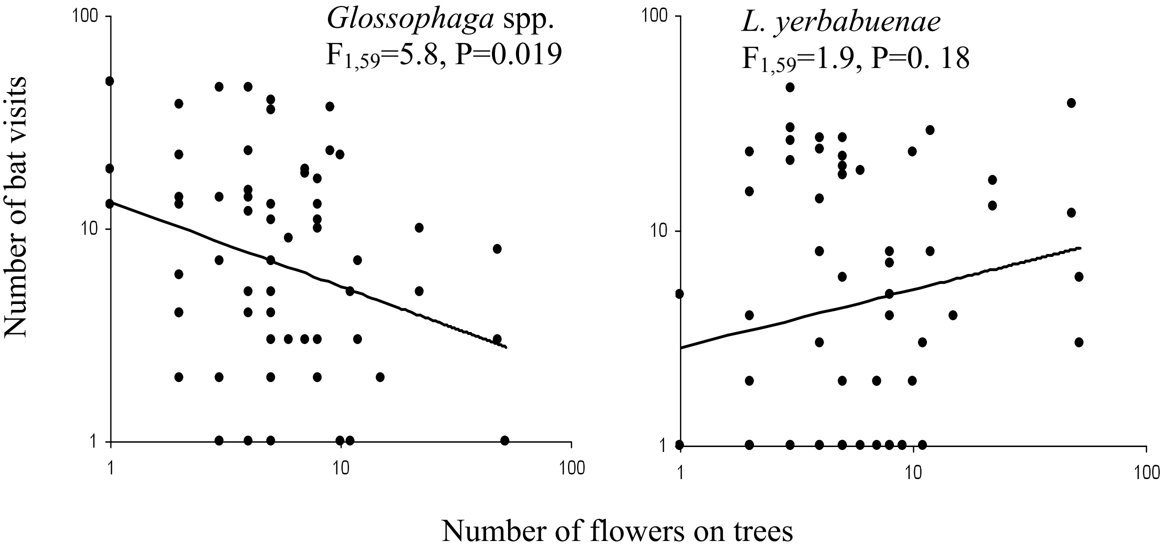

Data proceed from 4- to 5-hrs long infrared video recordings from 60 trees with flower numbers varying from 1 to 52 (mean= 8.9±11.8), in Chamela-Cuixmala Biosphere Reserve, Jalisco, Mexico. *Glossophaga* species could not be distinguished, but *G. soricina* is by far the most abundant of the two locally occurring *Glossophaga* species (unpublished data derived from [1]).

1. Stoner KE, Lobo JA, Quesada M, Fuchs E, Herrerías-Diego Y, et al. (2007) Efecto de la perturbación del bosque en la tasa de visitas de murciélagos polinizadores y sus consecuencias sobre el éxito reproductivo y el sistema de apareamiento en árboles de la familia Bombacaceae. In: Harvey CA, Sáenz JC. Evaluación y conservación de biodiversidad en paisajes fragmentados de Mesoamérica. INBio, Heredia, Costa Rica. pp. 351-372.
